# St. George's Hospital: The Special Court of Governors

**Published:** 1914-02-21

**Authors:** 


### ST. GEORGE'S HOSPITAL.

### THE SPECIAL COURT OF GOVERNORS.

A very fully attended meeting of Governors of the hospital was held in the Board Room on Thursday, February 12. Lord Greville was voted to the chair, and after the Chaplain had opened the meeting with prayer, the Secretary, Mr. Wingrove, read the minutes of the Court and of the committees.

### A Right of Every Governor.

Before the minutes were confirmed Sir Henry Burdett pointed out that those relating to the House Committee of the 11th instant were incomplete, owing to the omission of an important letter from the solicitors to the hospital which stated that in their view the opinion given by Mr. George Cave and Mr. James Rolt, the eminent counsel, to the effect that every Governor of St. George's Hospital under the laws has the right to be supplied on application with a printed list containing the names and addresses of the Governors was correct. The letter was sent for, read, and ordered to be entered on the minutes accordingly.

Mr. H. W. Hewitt next raised a point arising out of the minutes: that if the minutes as read were confirmed the effect would be that the Governors would pass a vote of censure on themselves if they sanctioned the resolution of the House Committee regretting the action of the Court on December 19. The Chairman ruled that Mr. Hewitt could raise the point later, but that the only matter before the Governors at the moment was to confirm the minutes as they stood as being correctly entered.

### A Committee on the Laws to be Appointed.

The Chairman: We now come to special business. The first item is that printed on the agenda as No. 1. That is the motion. There is an amendment to be moved by Sir Henry Burdett. I will read the amendment to you in case some of you have not got it: "That power be hereby given to the House Committee to appoint, at their discretion, a Committee of Governors to consider the laws of the hospital, to report whether any, and what, alterations should be made therein, and if they think fit to obtain legal assistance."

Sir Henry Burdett: On the notice which was sent out the wording of a resolution which has been included in the special business of this Court on a requisition from the Governors to the House Committee appears. At a meeting of the House Committee yesterday it was pointed out that one of the most important things which St. George's Hospital has to carry through, and carry through quickly, it is hoped, is the amalgamation of this hospital with Westminster Hospital. If we were to proceed to appoint a Committee of Governors to-day to go into the by-laws if they had completed their work or during the time they were engaged upon it, it is possible an agreement might be come to with the Westminster Hospital. Then the whole business would have to be gone into over again. It was therefore felt that it was better business, and more in order, that we should alter the resolution to the terms which you have just heard read. The effect of this will be to give power to the House Committee to appoint, at their discretion, that is when the time arrives in relation to the negotiations with Westminster Hospital, to appoint a Committee of Governors to consider the laws of the hospital, and to deal with them in conjunction with Westminster Hospital. I presume that the Committee of Governors will be a joint committee of the two hospitals, and in that way I hope we shall get a new set of laws which will commend themselves to everybody and be a great source of strength to this institution and to the institution with which we hope to amalgamate. I think it is most desirable that this hospital shall secure unanimity to-day in this matter. I have therefore to move: "That power be hereby given to the House Committee to appoint, at their discretion, a Committee of Governors to consider the laws of the hospital, to report whether any, and what, alterations should be made therein, and if they think fit to obtain legal assistance." I hope the committee will be small—I should say three, certainly not more than five Governors—and I hope every one of them will be gentlemen who have some knowledge of the drafting of laws and some knowledge too of the administration of hospitals. I have much pleasure in moving that amendment.

Mr. Stuart de la Rue: I second it.

Mr. F. J. Frankau: Might I ask one question on it? I take it that the word "Governors" includes members of the House Committee. It might be intended here, but I do not know whether it is the intention that the House Committee are to name Governors other than themselves.

Sir Henry Burdett: My answer to that would be that, as Sir George Jessel, when Master of the Rolls, declared, every extra word in a document amounts to a word of limitation. That ruling will apply here. We are trusting the House Committee; we wish to trust them. We want the House Committee to think of St. George's only, and if Westminster Hospital amalgamates, we want them to think of the two hospitals. So we will leave the House Committee with perfect confidence that they will appoint the very best men they can find from outside their own number or with some of their own members, so that every person selected may possess the special qualifications.

The Chairman: You have heard the amendment moved and seconded. I do not know whether there is any gentleman who wishes to speak upon it before we vote. If not I will put it.

Carried *nem. con.*

### An Unfortunate Misunderstanding.

The Chairman: The next item on the special paper is No. 2: "To consider and, if approved, to move a resolution regretting the action of certain Governors who, after bringing charges against Mr. West, as treasurer, and the medical staff, declined to abide by the result of an independent inquiry which the President proposed to institute and which the House Committee had welcomed." To that I understand Mr. Laurence Hardy wishes to move an amendment.

The Right Hon. Laurence Hardy, M.P.: Lord Greville, I desire to move an amendment to the resolution which stands on the paper, and I will give you the terms at once, so that you may understand the reason why I move it instead of the motion as it appears on the printed paper. I desire to submit this afternoon to the Court this resolution: "That the Court much regret that an unfortunate misunderstanding should have given her Royal Highness Princess Christian the impression that an objection had been raised to her Royal Highness's proposal to nominate an independent committee to investigate and report on the charges brought against the management of the hospital at the meeting of December 19, and upon the causes leading to the resignation of the two treasurers, and desire to place on record their unreserved acknowledgment of the right of the President to institute such an inquiry." The amendment differs from the resolution in omitting any reference to personalities. I think we have arrived at the position when we ought, if possible, to put absolutely aside the past and see if we cannot advance to unity and concord, and with that to the benefit of the hospital in the future. And, therefore, I think it would be only pursuing a vain task if we were to move a resolution to-day which gives any opportunity for reopening the feuds which, I am sorry to say, have been to some extent going on during the last few months. I speak comparatively impartially, for I only joined the House Committee in June, and I have not been able to attend very frequently, I am sorry to say, and therefore I am still outside the cognisance of the causes, to a great extent, which have unfortunately, I think, led to friction during these months. At all events, my sole aim and desire is that we should put all that in the background; but I do not think we could possibly have met here to-day without at all events alluding to the subject which is the main matter in the resolution which I move.

### The Resignations.

We have to face the facts as we have heard to-day in the minutes, the deplorable circumstance, of the resignations of our President and Vice-President, her Royal Highness Princess Christian and Princess Victoria, mainly on the ground that the authority of the President has been resisted. I wish to place clearly on record my opinion that her Royal Highness the President was perfectly correct in the attitude she adopted. She, as our President, is bound to look after the interests of the hospital. She had information supplied to her which impelled her to ask for an inquiry, an inquiry into circumstances which were stated openly at the Court here, and also into the causes which led to the resignation of Lord Plymouth and Mr. West. I think as President she was bound to consider her position, and, if possible, to adjust the matter. She sent that request—or perhaps it was more than a request—but she was justified as President in doing it. She sent forward her request for an inquiry to the House Committee, and the House Committee approved of it. Unfortunately, after that information she conceived, I think through a misunderstanding —and that is why I put it in my resolution—she conceived that her authority was contested to some extent within the hospital, and that impelled her to take the further step of withdrawing the inquiry and sending in her resignation, which, notwithstanding the action of the House Committee in endeavouring to persuade her to postpone it, has been definitely carried out. I do feel that we owe it to her Royal Highness, now that we are met together, to forward her a resolution acknowledging that she had the right to demand an inquiry, that there was matter to inquire into, and regretting that, through a misunderstanding, that inquiry has been given up, and we have suffered the great loss, which undoubtedly is a great loss, of the Royal patronage and help which we have had from Princess Christian and her daughter; and I think we could not omit to do that. The resolution keeps to the main point in view, and at the same time avoids every personal reference. I think it will be fortunate if we can get through this afternoon without any reference to the personal matters which have prevailed in the past. I hope that we may receive this resolution, if possible, unanimously, and I hope you may receive it without that debate which undoubtedly would have arisen if it had been moved in the original terms upon the paper. I trust everybody now is prepared to go forward and to make the best of the situation, and in that I am sure we shall do best not to go into any of these matters which I know there is too much reason to suspect would be raised in connection with these disputes of the past. We have undoubtedly suffered very considerable loss—I may say we have lost in the way of having suffered even considerable discredit in the eyes of the public—by having our dirty linen washed in public as we have lately. Let us now join hand in hand to make sure that we set our house in order so that we may be able to carry on the work of the hospital without any disagreement among those who manage it. I would, therefore, make a most earnest appeal that my resolution may be accepted in the spirit in which I am proposing it, and that without evading an extremely difficult subject we may, by accepting the words I have proposed, at all events not do any injustice or bring up any personality in connection with anything that has passed away. I beg to move that.

Mr. F. J. Frankau: The Right Hon. Gentleman who has moved this has moved it in words so apt and admirable that I do not think I can usefully add to them. I beg to second the motion.

### Mr. West's Decision.

Mr. A. W. West: May I rise and say how heartily I endorse the words which Mr. Laurence Hardy has said? I am the more particularly anxious to do this because I was the person around which much centred in December last. If I may be allowed to, I would say I wish to forget everything which took place then. I hope I may carry with me the unanimous opinion of this room, that we may all try to forget what took place as speedily as possible. I make that appeal to everybody. I believe the best course I can adopt is to cease entirely to have anything further to do with the affairs of this hospital, and I hope it will tend to bring back peace to the hospital. But there are two things which took place on December 19 which I should like to refer to, because they are financial matters; and what has gone forward to the public may give rise to misapprehension. Mr. Harrison said we were spending £18,000 a year above our income. Our friend Mr. Harrison has made some mistake. It is not the case, if he meant it in that sense, that we are spending £18,000 a year above our income.

Mr. Harrison: I took the amount from the amount which was published in the paper a week before that.

Mr. West: If you take the figures over ten years you will find we are spending £3,300 a year above our income. That is the actual figure. If you come to the capital account and you allow depreciation of stock for a period of ten years, you will find we have somewhat more capital than we had ten years ago. So £3,300 a year has been spent above our income. There is a surplus from other legacies. The other point is from a remark of Sir Henry Burdett's. He said that there was a loss of subscriptions to the hospital of £1,500 a year compared with ten years ago. Perhaps he did not realise that the figures he quoted, in the year 1902, show an increase from the special appeal here, and in that one year it went up £1,100 at a sudden bound, and it would have been more fair if he had taken the year before that, 1901. He would then have found that the total loss from subscriptions is £300 a year, not £1,500. Those are the only points I wish to put right. I am willing to do all I can, and I hope there will be no further discussion upon anything which took place on December 19.

The Chairman : Ladies and Gentlemen, you have heard the amendment which has been proposed by Mr. Laurence Hardy, and seconded by Mr. Frankau. Is it your pleasure to approve it?

Carried unanimously.

### Votes of Thanks to T.R.H. Princess Christian and Princess Victoria of Schleswig-Holstein.

Mr. Harrison : If I shall be in order I should like to make an addition to the third resolution which is put down here, the vote of thanks. I think it would be highly desirable and right, and I think we should not be doing anything like our duty if we did not, to move a vote of thanks to our late President, Princess Christian, and our late Vice-President, Princess Victoria, for the services which they have rendered towards the hospital. I am sure we all much regret the loss of their Royal Highnesses from being our President and Vice-President, and, as I explained on one or two occasions before, I think it is a great loss generally to our hospital that we have not been able to retain their services. I might add also what has been suggested to me, a suggestion whether we might ask them to continue their interest in the hospital in future, although they may not act as President and Vice-President. I beg to move that that vote of thanks be sent to them.

Mr. Laurence Hardy : I second Mr. Harrison's resolution.

Carried unanimously.

### Votes of Thanks to the Retiring Treasurers.

The Chairman : The next item on the paper is : "To consider, and, if approved, to move a vote of thanks to the retiring treasurers for their services to the hospital, and that they be asked to accept the position of Honorary Life Governors."

Dr. Cahill : My Lord and Gentlemen,—I propose this resolution in the form in which I have handed it up to the Chair. In includes thanks to both treasurers who have sent in their resignations. The Earl of Plymouth has been treasurer of the hospital since February 1904, that is, ten years ago, and Mr. West has been deputy-treasurer thirteen years. It has always been the custom hitherto at St. George's to have two treasurers : the first treasurer has been a nobleman of high position, whose name has been a guarantee to the public as regards the administration of the hospital, but he has never been called upon to take any active part in the hospital administration. We have always had, on the other hand, as second treasurer some gentleman who has undertaken to look after the executive management of the hospital as well as the administration of its funds, and that work has always taken up a great deal of time on the part of the gentleman who undertook it. I have heard it said that perhaps a treasurer may devote too much time to the work, or may take upon himself more than the necessary amount of care in the management of the institution. I would like to point out to those who have said that—because I have heard it said recently—that our working treasurer has always been expected to do, and has done, a great deal of work in the details of administration. The fact that Mr. West was appointed when Mr. Holmes had not retired shows that the treasurer is expected to do a great deal. Although Mr. Holmes was able to supervise the administration to a certain extent, the Board of Governors considered that a younger man was wanted to help him in the constant, almost daily, attention which the treasurer is expected to give to the details of administration of the hospital. In the case of Mr. West the work which he has carried out for the last thirteen years has admittedly been carried out with the best results in regard to efficiency and his administration in St. George's Hospital. Recently there has been a lot in the press, owing to various dissensions which have taken place, about the management, or mismanagement, at St. George's, and I think it cannot be too emphatically pointed out that, whatever disagreements may have arisen over certain matters of detail, there has never been for a moment any pause in the activity or efficiency of St. George's Hospital and its work in looking after the sick poor. At the present moment the work which is going on in the wards and the out-patient departments of this hospital is being carried on as efficiently as it could be in any institution in this kingdom, and no differences which have taken place in this Board Room have interfered with the management of St. George's in its great object, the care of the sick poor. And that cannot be too strongly emphasised. Its efficiency has been raised during the last thirteen years, under Lord Plymouth and Mr. West, to a point which it had never reached before. I do not wish to belaud the present standing of the hospital, but medical science has advanced and knowledge in medical nursing has advanced, and in those advances St. George's has taken a very prominent part. It is impossible, where everyone has been working for the same object, to attribute the good work to any one man, but those who are familiar with the work which has been carried on during recent years will agree that the treasurers, especially the working treasurer, have taken a very leading part in promoting the efficiency to which the hospital has attained as a beneficent medical charity. There are so many details into which one might go, but speaking as an old Governor of many years' experience, and one whose connection with the hospital has been fairly intimate from a Governor's and a medical point of view, it is marked on entering the hospital and going about it the happiness of patients and nurses, showing that the best relations exist between them as to the comforts of the patients and the care and attention of the nurses. And much of this is brought about by the greater care given to training nurses compared with ten or thirteen years ago. There is also the question of properly cooked food, and in all those respects we have markedly advanced. During the tenure of office of the late treasurers we have also instituted various advances in the work of the hospital, notably in the provision of a radiological and electrical department, which was long considered a necessity by many Governors, and for which it was difficult to find space or room. Mr. West had more to do with that than anybody else to enable us to have that department, and the efficiency of that department was recognised recently when a daily paper, when making a collection to supply radium, allocated to St. George's Hospital £1,000 for its purchase, showing that the electrical department had already obtained a standing in the medical world.

### Reforms of Recent Years.

During recent years considerable reforms have been made, notably in the reorganisation of the almoners' department, which has not only to do with the right people receiving the charity of the hospital, but assuring that those who are under the care of the hospital may have every means of attention out of doors and during convalescence, and that was also specially put forward by

Mr. West. During that time also we have established the nursing institution for the supply of nurses from St. George's Hospital to outside patients. That does not touch the work of the nurses in connection with patients in the hospital, but it does much to maintain the position of St. George's and secure sympathy with it, and to secure a spirit of satisfaction among the nurses working in the hospital that they know they may have a chance of becoming employed outside.

### An Inside View.

I wanted to refer to these changes in the administration which have been brought about during the tenure of Lord Plymouth and Mr. West, but it would be affectation on my part to ignore the circumstances which have recently taken place. I do not wish to raise unpleasant personalities or to detract from the spirit of unanimity which has pervaded this meeting, but I may be allowed, without touching deeply on the matter, to say that the unfortunate position in which we find ourselves through the retirement of Mr. West has been largely due to misunderstandings, and largely on the part of Mr. West himself, and that not by Mr. West's fault, but because, owing to circumstances, the views of the Governors and of the House Committee had been conveyed to Mr. West in a form which led him to believe his position was different from what it actually was. I think it right to say as much as that, because otherwise the statements made as to the position of Mr. West with regard to the House Committee on December 19 might very well be very much misunderstood outside. The idea was put abroad, and these things have been repeated in newspapers outside, that Mr. West had offered himself on his retirement from the treasurership to obtain a paid situation in the service of the hospital. As a member of the House Committee, who has been in intimate connection with what is going on, I say that any such insinuation or suggestion is entirely contrary to the facts. Mr. West, after serving thirteen years as treasurer and devoting the best working years of his life to this hospital, had, to his great regret—I know it from his having mentioned the matter to me a fairly long time ago—come to the conclusion that he could not give up to the hospital the time he was giving up to it without damaging his own material interests to an extent which he could not afford to do.

### The Salaried Post.

In other words, Mr. West started the treasurership as a young man, and having won a foremost position in a scientific profession, found, after thirteen years' work in this hospital, that he could not advance in the work of his profession as a man of his standing and ability had a right to expect. He had been sacrificing his livelihood and his professional position to the work he has been doing here. That is a perfectly plain statement of fact and it needs no comment. Mr. West said he would retire from the treasurership, and last year he intimated that, and intimated it in a form which would allow the House Committee time to consider what steps they would take. The idea to ask Mr. West to occupy a salaried position originated before he announced his retirement from the House Committee and did not originate with Mr. West. So long ago as the beginning of last June I was asked what I thought would happen if Mr. West gave up the treasurership, which was then already a matter of common talk. I said I did not know, but would it be possible to persuade him to carry on the work, at all events until the period of amalgamation has gone by, or the period of sale, in some salaried department? And that was the first intimation I had that there was any idea that Mr. West should be asked to enter the hospital temporarily as a paid official. That came from a Governor independently of Mr. West; it came from one who was thinking only of the interests of the hospital and the medical school. And there came a feeling that, with the experience Mr. West had of the administration, with the knowledge he had of the circumstances and the conditions of the hospital, and the work he did towards the disposal of the site and the effort to bring about a satisfactory bargain, we should seriously feel the loss of that man when it came to the important work of selling the site and moving the hospital elsewhere, more especially as it happened that in Mr. West we have for business of that sort a man with special technical experience as a practical architect and surveyor. So that this, in working for the hospital in these matters, was a very important matter. I know that that idea originated amongst Governors and members of the House Committee independently of any movement on the part of Mr. West. What happened?

### The Secretaryship.

A suggestion was made that a committee should be appointed to consider this question as to whether, on Mr. West's retirement, anything could be done to bring about the retention of his services, at all events during this period of transition. A sub-committee sat on that question, and it reported that it did not think it in any way desirable to alter the conditions under which the treasurership of the hospital had hitherto been held, or to offer a salary to a paid treasurer, but they considered that in some other way Mr. West's services might be retained. Upon that a suggestion was made, and it came entirely from members of the House Committee, supported by outside Governors, and not at the instigation of Mr. West, that Mr. West might be asked to occupy the position of secretary, in order to carry on the work he was doing, and to aid us through the difficult times which were to come. Another sub-committee was appointed to consider that question, and it reported to the House Committee unanimously in favour of the suggestion, and the House Committee accepted that report unanimously. When this report of the sub-committee and the House Committee came before the Court, things were said and things were subsequently reported in the press which did not give a true interpretation of the facts which I have narrated. Mr. West came before that Court having, as far as he knew, behind him the request of a body of men responsible for the management of the hospital, who pushed him forward and asked him to undertake certain work, and to be paid for it. They did not ask him out of love for him, but they they thought he was practically the man running the business of this hospital, and so that to a large extent he was better able to look after the work in a paid position than any man they could get from outside.

### A Misunderstanding.

That is the position he accepted, but he did not know that at the time these reports were unanimously accepted by the House Committee they were already being objected to. Mr. West did not know that when the unanimous recommendation came before the Court it would be opposed, after the fashion we heard on that day. But much of that trouble arose from misunderstanding on the part of Mr. West himself. I do not think Mr. West was to blame for that misunderstanding. It was thrown upon him. He was asked.

unanimously as he thought, was assured unanimously of the support of the management to undertake that position, and under those conditions he gave way to the wish of a large number of the Governors. I am certain that if it had been conveyed to Mr. West that the approval and recommendation which was recorded by the House Committee was not unanimous, he would have been the last to allow himself to be brought forward. It is due to Mr. West that it should be explained that the position he found himself in was one which was thrown upon him, and was not sought by himself; he thought he had the unanimous support of the governors. That shows that much of the unpleasantness which arose and the attack on Mr. West might have been avoided if the position which Mr. West occupied had been treated by those responsible for putting forward these recommendations with a little more genuine straightfordwardness and straight dealing. I do not wish to detain the Court longer, but I will conclude with the resolution which I have handed in, proposing a vote of thanks to Lord Plymouth and Mr. West, the treasurers, and that both gentlemen be asked to accept the position of Life Governors of the hospital.

### The Medical Staff's View.

Mr. G. R. Turner: As the senior member of the staff, I would like to second that resolution, and express here publicly how much during Mr. West's tenure of office the staff have been indebted to him for the constant and impartial way in which he has always looked, not only to the benefit of the patients, but of everyone connected with the hospital—all the officials. Of course, now and then during the thirteen years it is only likely that there have been acute differences of opinion about this, that, and the other, and Mr. West, who has been very energetic indeed, has on occasion had differences with us; but I can speak with knowledge of a good many treasurers, and I can say honestly that nobody has done so much work about the hospital, and done it in so thorough a way, as Mr. West. Now, Sir, Mr. West has to-day said that all personal matters, as far as he is concerned, are things of the past. I may say, in the same spirit of peace, that all personal matters with reference to the staff, and the accusations—the untrue accusations—made against them at the last Court of Governors also I shall not answer, because I think it well, for peace, that we should, although not acknowledging any of the accusations made against the staff, we should not introduce into this meeting, which has been one of harmony hitherto, anything which may upset the good feeling which I hope is going to prevail in future amongst all connected with St. George's Hospital; but I should like to take the opportunity of endorsing what Dr. Cahill said, that it was not Mr. West's suggestion that he should be a salaried official of this hospital. That I know for a fact. I could give details, but we have already been here so long that I will not.

### The Medical School.

And, again, I should like to say that it is not a true thing that Mr. West has in any way injured the medical school. The medical school at the present time is in a more flourishing condition than it has been since it ceased to be a school which taught the elementary subjects and has been only a clinical school. Mr. West has done nothing but good to and for the school. With reference to the work that the staff has done with Mr. West, and especially in the more or less critical times that are past now, I am glad to say, when there was a difference between the King's Fund and this hospital, Mr. West, it is true, had the moral support of this hospital through that controversy, but he did not rely on the votes on the part of the House Committee to carry his policy; and I may say that at the two critical meetings, on April 12, 1911, there were twenty members present at the House Committee, four only of the staff. The resolutions supporting his policy were carried by thirteen to two against. At another critical division, on February 21, 1912, again his policy was endorsed by the House Committee: nine voted for, three against, and no member of the medical or surgical acting staff voted at all. I mention those facts, Sir, simply because it has been put about, and to Mr. West's possible detriment, that he, in return for our support, has done this, that, and the other. I do not want to re-open questions which are, I think, buried, but I would say Mr. West has not run this hospital simply and solely by the support given to him by the medical staff. The medical staff have always tried to support what they believe to be for the good of the hospital; and the medical staff feel the resignation of our President, Princess Christian, and even of our treasurers, more than, perhaps, the lay Governors, who can come to St. George's, and if they do not approve of what is going on can shake the dust of St. George's off their feet and go elsewhere. But we, the medical staff, have got to carry on the work of looking after the sick poor here, whatever divisions there may be amongst the laity of our governing body. And, therefore, we feel very strongly anything like the resignation of our President, or the resignation of our treasurers. We cannot get away from the hospital. The great Duke of Wellington said: "The King's Government has got to be carried on"; the work of caring for the sick poor has got to be carried on at Hyde Park Corner, and we cannot get away from it. Therefore it is to the interests of the medical staff and the medical Governors more than any others that the Governors of the hospital should have the interests of St. George's at heart. While Mr. West has been treasurer, I think I can say, on behalf of my colleagues, that in our opinion that has been the case.

The resolution was carried unanimously.

### Thanks to Lord Greville.

Mr. West: There is one more vote which I want to move, but before I put it to you I want to thank you sincerely for your kind vote of thanks. It has been nothing but a pleasure, and always will be, to have worked during these years for St. George's Hospital. I desire to move a vote of thanks to Lord Greville for taking the chair to-day, and also for taking us so well through a critical period in this hospital's history.

### Carried by Acclamation.

The Chairman: I thank you for this vote. As I felt that there were members who could take the chair better than myself I can only say from the bottom of my heart I am glad that this meeting has gone so well, and I hope it has closed what has been, certainly for many of us, a very unpleasant time with regard to St. George's Hospital.

